# GP wellbeing during the COVID-19 pandemic: a systematic review

**DOI:** 10.3399/BJGP.2021.0680

**Published:** 2022-03-22

**Authors:** Laura Jefferson, Su Golder, Claire Heathcote, Ana Castro Avila, Veronica Dale, Holly Essex, Christina van der Feltz Cornelis, Elizabeth McHugh, Thirimon Moe-Byrne, Karen Bloor

**Affiliations:** Department of Health Sciences, University of York, York.; Department of Health Sciences, University of York, York.; Department of Health Sciences, University of York, York.; Department of Health Sciences, University of York, York.; Department of Health Sciences, University of York, York.; Department of Health Sciences, University of York, York.; Department of Health Sciences, University of York, York.; Department of Health Sciences, University of York, York.; Department of Health Sciences, University of York, York.; Department of Health Sciences, University of York, York.

**Keywords:** coronavirus, COVID-19, general practice, mental health, systematic review, wellbeing

## Abstract

**Background:**

Doctors’ organisations in the UK have reported worrying levels of work-related stress and burnout in the GP workforce for some time, and the COVID-19 pandemic has presented clear new challenges.

**Aim:**

To synthesise international evidence exploring the impact of COVID-19 on primary care doctors’ mental health and wellbeing, and identify risk factors associated with their psychological wellbeing during this time.

**Design and setting:**

Mixed-methods systematic review.

**Method:**

Six bibliographic databases, Google Scholar, and *MedRxiv* were searched on 19 November 2020 and 3 June 2021 to identify studies of GP psychological wellbeing during the pandemic. Reference checking was also conducted. Two reviewers selected studies, extracted data, and assessed the quality of studies using standardised tools. Heterogeneity in outcomes, setting, and design prohibited statistical pooling; studies were combined using a convergent integrated thematic synthesis.

**Results:**

Thirty-one studies were included. Multiple sources of stress were identified including changed working practices; risk, exposure, and inadequate personal protective equipment (PPE); information overload; pandemic preparedness; and cohesion across sectors. Studies demonstrated an impact on psychological wellbeing, with some GPs experiencing stress, burnout, anxiety, depression, fear of COVID-19, lower job satisfaction, and physical symptoms. Studies reported gender and age differences: women GPs had poorer psychological outcomes across all domains, and older GPs reported greater stress and burnout. Use of outcome measures and reporting practice varied greatly.

**Conclusion:**

This review of international evidence demonstrates that the COVID-19 pandemic has adversely affected GPs’ wellbeing around the world. Further research could explore gender and age differences, identifying interventions targeted to these groups.

## INTRODUCTION

Doctor burnout has been described as a global crisis^1^ affecting the quality of patient care^2^^–^^4^ and the sustainability of healthcare systems.^5^ International literature highlights growing problems with chronic stress and burnout that threatened the mental health of doctors working in primary care settings before the COVID-19 pandemic.^6^^–^^12^ In the UK, 37% of GPs surveyed in 2019 reported an intention to leave direct patient care,^13^ and researchers have estimated a shortage of 2500 GPs, projected to increase to 7000 within 5 years if current trends continue.^14^

The COVID-19 pandemic has presented additional challenges for primary care doctors around the world, including rapid change, risks of infection, remote working, pent-up demand, reductions in face-to-face patient care, and vaccination delivery. Research from earlier epidemics and emerging during the COVID-19 pandemic demonstrates a negative impact on clinician psychological wellbeing.^15^^–^^19^ A 40% increased use of mental health support services has been reported during the pandemic (across UK health professional groups).^20^

While there has been a tendency for research to focus on hospital roles,^21^ there is now a need to synthesise evidence and explore factors associated with primary care doctors’ mental health and wellbeing during the pandemic.

## METHOD

Cochrane guidance for conducting systematic reviews^22^ was followed and the study was registered and a protocol published (PROSPERO ID: CRD42020225680).^23^ The PRISMA checklist^24^ was used to ensure the transparency of reporting.

### Search strategy

Six bibliographic databases (MEDLINE, Embase, PsycINFO, Science Citation Index, Social Science Citation Index, and Emerging Sources) were searched for GP wellbeing during the COVID-19 pandemic. Owing to the current nature of the topic, Google Scholar and *MedRxiv*, a preprint service for health research, were also searched (see Supplementary Appendix S1 for full searches). No date or language limits were applied at the search stage. A date limit (2019 onwards) was applied once the records were entered into Endnote (version 20) to capture studies measuring outcomes during the pandemic. Reference lists of included studies were also searched. The initial search was undertaken on 19 November 2020; this was updated on 3 June 2021.

### Inclusion criteria

Studies in any country examining the impact of the COVID-19 pandemic on measures of primary care doctors’ psychological wellbeing, stress, and burnout, with absenteeism and markers of workforce retention as secondary outcomes, were included. Studies solely exploring doctors’ infection rates were excluded. International variations in terminology, for example, doctors working in general practice/family practice/primary care were used; for simplicity, in this article all are referred to as ‘GPs’. Non-English language studies and those including multiple health professional groups that did not present the results for GPs separately were excluded. Searches were not limited by study design in this mixed-methods systematic review, but only empirical research was included; editorials and purely descriptive articles were excluded. Studies rated as high risk of bias were excluded from the synthesis.

**Table:** How this fits in

Many GPs have reported stress and burnout over recent years, which is potentially damaging not just to doctors themselves but also to patients and healthcare systems. The COVID-19 pandemic has presented new challenges and there is a need to evaluate the impact on GP wellbeing. This review synthesises the international evidence base exploring primary care doctors’ psychological wellbeing during the pandemic. Studies have highlighted multiple sources of stress during this time and report experiences of stress, burnout, anxiety, depression, fear of COVID-19, reduced job satisfaction, and physical symptoms. Gender and age differences may warrant further research to identify interventions targeted to the needs of specific groups.

### Selection of studies

The results of each search were entered into an Endnote Library and duplicates removed. Two independent reviewers screened resulting records using titles and abstracts. Two of four reviewers screened the full text of all studies deemed potentially relevant and any disagreements were resolved by a third reviewer.

### Data extraction

One of three reviewers extracted data using a pre-piloted data extraction form, cross-checking a 20% sample to ensure consistency. Information was extracted regarding study design, sample size, sample characteristics, and primary and secondary outcomes.

### Quality assessment

The quality of identified reviews was assessed using the Joanna Briggs Institute (JBI) Checklist for Analytical Cross Sectional Studies^25^ and the Critical Appraisal Skills Programme (CASP) quality checklist^26^ for observational and qualitative studies. Two researchers independently quality assessed the included studies, with disagreements resolved by a third reviewer. Studies were excluded if ≥4 categories were rated as inadequate on the JBI tool^25^ or if qualitative studies were rated as being ‘invaluable’ using the CASP tool^26^ because of significant issues in the design and conduct of the study.

### Data synthesis

Pooled analysis (random effects) was used to summarise age and gender data across studies. Where age groups were reported, the average age was estimated using the midpoint and frequency of the age groups. Data did not meet the requirements for statistical pooling of outcomes because of heterogeneity in outcome measures, study designs, and healthcare settings.

As this mixed-methods systematic review included both quantitative and qualitative study designs covering broadly similar topics, a convergent integrated approach was undertaken based on the JBI guidance for mixed-methods systematic reviews.^27^ This involved a form of narrative synthesis whereby quantitative data are described alongside qualitative findings under common themes or categories.^27^ NVivo (version 12) software was used to manage and sort data, following a process of thematic qualitative synthesis that moves from initial ‘free coding’ through to descriptive and then more analytical themes.^28^ This process was iterative, with codes and themes refined and developed throughout the analysis process, through consultation among the wider research team.

## RESULTS

### Search results

**Figure 1.**
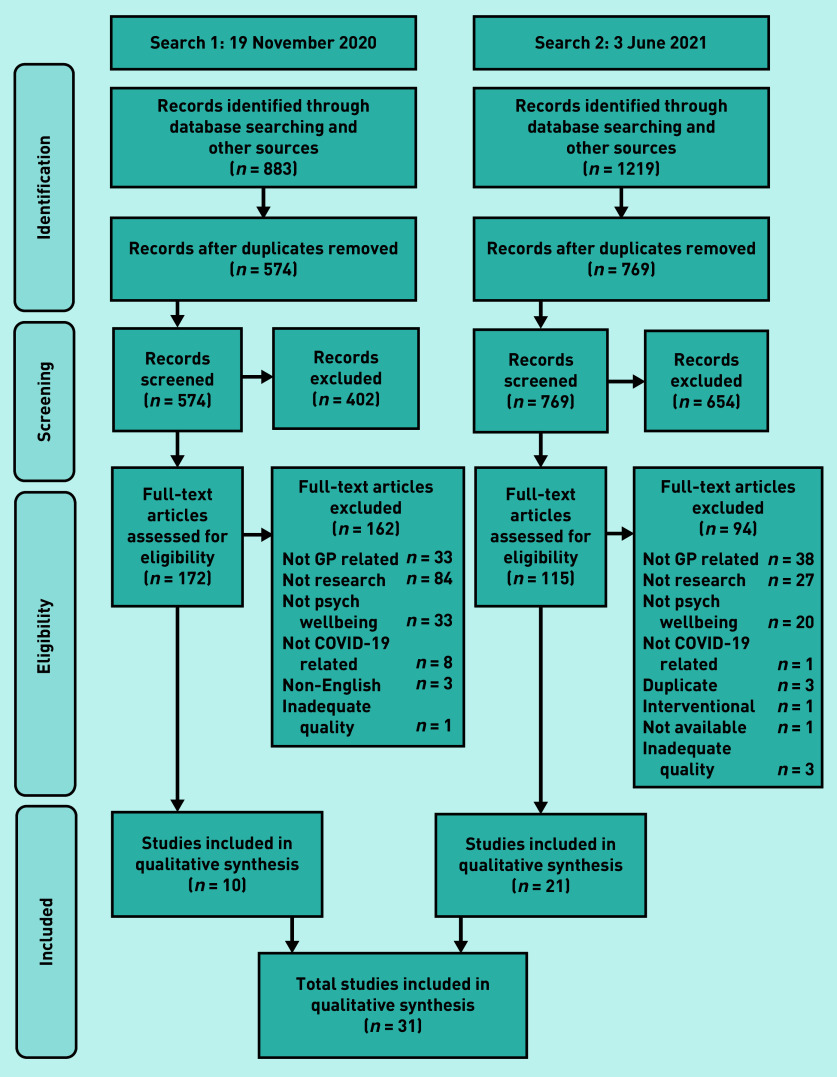
*Flow diagram for included studies. Psych = psychological.*

In total, 2102 studies were retrieved from databases and hand searching. There were 759 duplicates that were removed, and 1056 studies were excluded by screening the titles and abstracts. This resulted in 287 full texts being screened and 31 studies^29^^–^^59^ being included overall (Figure 1).

### Description of studies

Characteristics of the included studies are presented in Supplementary Table S1. Studies were dispersed geographically, with the largest numbers undertaken in Italy^32^^,^^42^^,^^46^^,^^52^ and China^35^^,^^50^^,^^51^^,^^59^ (Table 1).

**Table 1.** Summary characteristics of studies

**Characteristic**	***n***
**Location of study**	
Italy	4
China	4
Singapore	3
France	2
Colombia	2
UK	2
US	2
Australia	1
Croatia	1
Indonesia	1
Jordan	1
Oman	1
Portugal	1
Romania	1
Saudi Arabia	1
Spain	1
Turkey	1
Multiple countries	2

**Demographic, mean (95% CI), range**	
Age, years	42.4 (39.6 to 45.2),^a^ 26–55
Gender, % male	41.3 (34.6 to 48.5), 15–100

a *Based on 23 studies reporting sufficient information.*

There were 25 cross-sectional surveys,^30^^–^^33^^,^^36^^–^^50^^,^^52^^,^^54^^,^^55^^,^^57^^–^^59^ five qualitative studies,^34^^,^^35^^,^^51^^,^^53^^,^^56^ and one mixed survey and qualitative study.^29^ Several validated and some non-validated measures were used to assess outcomes. Sample sizes ranged from 86 to 1040 participants (median 330) for the studies with survey designs, and 11 to 80 for the qualitative interview studies (median 14). Demographic characteristics commonly reported by studies included age and gender (Table 1), with mixed reporting of other characteristics such as years of experience (Supplementary Table S1).

### Quality assessment

The quality of included studies was generally good (Boxes 1 and 2).

**Box 1.** Quality appraisal of cross-sectional surveys using the JBI tool^a^

**Author (year)**	**1. Were the criteria for inclusion in the sample clearly defined?**	**2. Were the study subjects and the setting described in detail?**	**5. Were confounding factors identified?**	**6. Were strategies to deal with confounding factors stated?**	**7. Were the outcomes measured in a valid and reliable way?**	**8. Was appropriate statistical analysis used?**
Amerio *et al* (2020)^52^	Yes	Yes	No	No	Yes	Yes
Alrawashdeh *et al* (2021)^29^	Yes	Yes	Yes	Yes	Yes	Yes
Baptista *et al* (2021)^41^	Yes	Yes	No	No	Yes	Yes
Castelli *et al* (2021)^46^	Yes	Yes	No	No	Yes	Yes
Di Monte *et al* (2020)^42^	Yes	Yes	No	No	Yes	Yes
Dutour *et al* (2021)^30^	Yes	Yes	Yes	Yes	Yes	Yes
Filfilan *et al* (2020)^49^	Yes	Yes	No	No	Yes	Yes
Gold *et al* (2021)^57^	Yes	No	No	No	Unclear	Yes
Gokdemir *et al* (2020)^33^	No	Yes	Partly	Partly	Yes	Yes
Jahan *et al* (2021)^40^	No	Yes	No	No	Yes	Yes
Lange *et al* (2022)^36^	Yes	Yes	No	No	Yes	Yes
Lau *et al* (2021)^31^	Yes	Yes	Yes	Yes	Unclear	Yes
Lau *et al* (2021)^54^	Yes	Yes	No	No	No	Yes
Lee *et al* (2020)^37^	Yes	Yes	No	No	Yes	Yes
Ortega-Galán *et al* (2020)^38^	Yes	Yes	No	No	Yes	Yes
Monterrosa-Castro *et al* (2020)^48^	Yes	Yes	No	No	Partly	Yes
Monterrosa-Castro *et al* (2021)^44^	Yes	Yes	No	No	Yes	Yes
Rossi *et al* (2020)^32^	Yes	Yes	Yes	Yes	Yes	Yes
Sitanggang *et al* (2021)^43^	Yes	Yes	No	No	Yes	Yes
Sotomayor-Castillo *et al* (2021)^58^	Yes	No	No	No	No	Yes
Stafie *et al* (2021)^47^	Yes	Yes	No	No	Yes	Yes
Taş *et al* (2021)^45^	Yes	Yes	No	No	Partly	Yes
Trivedi *et al* (2021)^55^	Yes	Yes	No	No	Yes	Yes
Tse *et al* (2020)^59^	No	Yes	No	No	Yes	Yes
Vilovic *et al* (2021)^39^	Yes	Yes	No	No	Yes	Yes
Zeng *et al* (2021)^50^	Yes	Yes	No	No	Yes	Yes

a *Questions 3 and 4 on the JBI were not applicable and are excluded here. JBI = Joanna Briggs Institute.*

**Box 2.** Quality appraisal of qualitative studies using the CASP tool

**Author (year)**	**Section A: Are the results valid?**						**Section B: What are the results?**			**Section C: Will the results help locally?**
	**1. Was there a clear statement of the aims of the research?**	**2. Is a qualitative methodology appropriate?**	**3. Was the research design appropriate to address the aims of the research?**	**4. Was the recruitment strategy appropriate to the aims of the research?**	**5. Were the data collected in a way that addressed the research issue?**	**6. Has the relationship between researcher and participants been adequately considered?**	**7. Have ethical issues been taken into consideration?**	**8. Was the data analysis sufficiently rigorous?**	**9. Is there a clear statement of findings?**	**10. How valuable is the research?**
Alrawashdeh *et al* (2021)^29^	Yes	Yes	Yes	Yes	Yes	No	Yes	Yes	Yes	Valuable
Gomez *et al* (2021)^34^	Yes	Yes	Yes	No	Yes	No	Yes	Yes	Yes	Valuable
Taylor *et al* (2021)^53^	Yes	Yes	Yes	Yes	Yes	No	Yes	Yes	Yes	Valuable
Wanat *et al* (2021)^56^	Yes	Yes	Yes	Yes	Yes	No	Yes	Yes	Yes	Valuable
Xu *et al* (2020)^35^	Yes	Yes	Yes	Yes	Yes	No	Yes	Yes	Partly	Valuable
Yin *et al* (2021)^51^	Yes	Yes	Unclear	Yes	Yes	No	Yes	Yes	Yes	Valuable

*CASP = Critical Appraisal Skills Programme.*

#### Quality of cross-sectional surveys

Using the criteria outlined in the JBI tool,^25^ sampling was well defined, as were study participants and settings in most studies (Box 1). Studies reported age and gender inconsistently or lacked reporting of wider characteristics. This was more common for studies reporting GP outcomes alongside other professional groups. Most studies used objective and validated measures, although some also developed measures specifically to answer novel research questions around the impact of COVID-19,^39^^,^^59^ which had not been validated owing to the timeframes.

Although statistical analyses were appropriately conducted across studies, very few studies considered confounders or used strategies to deal with these. Four studies did this^29^^–^^32^ and one study partly explored confounders.^33^ Inadequacies in reporting were problematic, for example, the most commonly used measure was the Perceived Stress Scale (PSS), measured in seven studies,^30^^,^^33^^,^^36^^–^^39^^,^^55^ but this was poorly reported at times and different versions were used.

#### Quality of qualitative studies

Assessment using the CASP tool^26^ found all studies involving qualitative methods provided a clear statement of aims and study methodology, and the methods were deemed appropriate to address the aims of the research (Box 2). All but one study^34^ used suitable recruitment strategies, and all studies were rated as collecting data appropriately and conducting sufficiently rigorous analyses to address the research questions. No studies described consideration for the effect of the relationship between interviewee and researcher. There was some ethical review in all studies, although for the majority there was limited discussion of the issues considered. One qualitative study^35^ met the CASP quality criteria, but lacked clear information about which type of health professional the quotations related to (since multiple health professional groups were included). The study authors provided this information on request.

### Thematic findings

**Figure 2.**
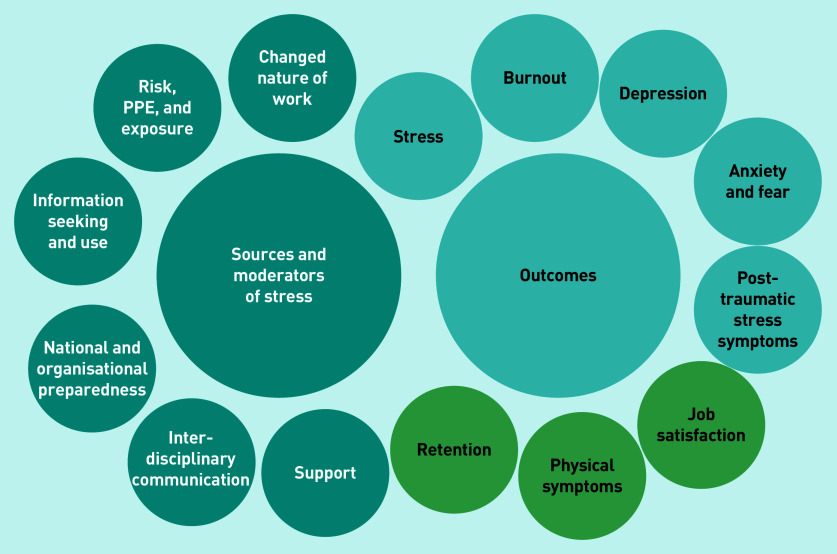
***Stressors, moderators, and outcomes relating to GP wellbeing during the COVID-19 pandemic.*** *^a^* *^a^* ***Light blue circles indicate psychological outcomes, while green circles indicate other outcomes. PPE = personal protective equipment.***

Findings were grouped into two overarching categories: 1) stressors and moderators; and 2) psychological wellbeing outcomes (Figure 2 and Supplementary Table S2).

### Stressors and moderators

Both qualitative and quantitative studies assessed sources of stress during the pandemic and these were grouped thematically as factors associated with the changing nature and quantity of GP work, risk and exposure, information seeking and use, organisational and national preparedness, and interdisciplinary communication. Support was seen as a moderator of stress. Further descriptions can be found in Supplementary Table S2.

### Psychological wellbeing outcomes

Reporting and measurement of psychological outcomes varied across studies, making comparisons across settings difficult.

Studies measuring stress placed GPs, on average, into ‘borderline’ or ‘stressed’ categories of the PSS^30^^,^^36^^–^^39^ and, using other stress scales, moderate-to-severe stress was reported in between 9.5% of GPs in Oman^40^ and 24.7% of GPs in Portugal.^41^ In terms of burnout, studies found the greatest difficulties related to emotional exhaustion, with 24.5% to 46.1% of GPs reporting high burnout symptoms relating to the emotional exhaustion components of the scale.^36^^,^^42^

Rates of anxiety ranged from 20% in Indonesia^43^ to 95% in Turkey and Colombia.^44^^,^^45^ Symptoms of depression were reported to a lesser extent, and ranged from 13% in Indonesia^43^ to 37% in Italy.^46^ Post-traumatic stress symptoms were reported in 10.6% of GPs in France,^36^ moderate-to-severe symptoms were reported in 45.2% of GPs in Croatia,^39^ and 32% of GPs in Italy presented with significant post-traumatic stress symptoms.^46^

#### Occupational groups

Among five studies of mixed groups of healthcare workers, primary care doctors reported higher levels of personal perceived stress,^37^^,^^38^ worse burnout scores (relating to lower ‘compassion satisfaction’ and higher ‘compassion fatigue’),^38^ worse depression scores,^39^ greater reporting of post-traumatic stress symptoms,^32^ and lower job satisfaction than other specialty groups.^29^

#### Gender

Seven studies report statistically significant differences in outcomes for female GPs, including higher stress levels,^30^^,^^36^^,^^38^^,^^39^^,^^47^ greater reporting of burden and burnout,^36^^,^^41^^,^^47^ greater reporting of anxiety,^48^ and higher mean post-traumatic stress symptom scores.^36^

#### Age

Older age was associated with higher stress levels in three studies,^39^^,^^49^^,^^50^ but in GPs in Colombia younger age predicted anxiety,^48^ and in Portugal greater levels of depression were reported in GPs aged <40 years.^41^ In this study of Portuguese GPs, increased length of time working as a doctor predicted higher burnout on items of the burnout scale relating to patient interactions.^41^

### Other outcomes

Four studies explored future intentions, reporting wide variations in plans to leave medicine, which were associated with general anxiety, particularly around infection risk.^30^^,^^31^^,^^48^^,^^51^ Two studies report that 7% of GPs considered leaving practice,^30^^,^^31^ and another^48^ found that these intentions were associated with anxiety around protecting family members.

Ten studies explored impact on physical symptoms and general quality of life.^41^^,^^44^^–^^46^^,^^48^^,^^50^^–^^53^^,^^58^ GPs reported migraines and headaches, tiredness and exhaustion, sleep disorders,^45^^,^^50^ and increased eating, drinking, and smoking.^44^^,^^48^^,^^51^ More severe insomnia was associated with depressive symptoms in GPs in Italy.^52^

GPs in the UK with symptoms of long COVID felt ‘let down’ and expressed frustration at the lack of support and recognition for the condition.^53^

## DISCUSSION

### Summary

The COVID-19 pandemic has necessitated substantial changes in primary care around the world; GPs rapidly changed working practices and managed evolving guidelines amid uncertainty and personal risk. This review of international literature highlights the difficulties that GPs have experienced across healthcare settings during the pandemic and shows there are high levels of work-related stress and burnout.^30^^,^^32^^,^^33^^,^^36^^–^^42^^,^^47^^,^^55^ Rates of anxiety and depression varied considerably across international settings, as did the use of tools to measure such outcomes. Studies also lack longitudinal or ‘pre-pandemic’ comparators, which makes drawing firm conclusions regarding the impact of COVID-19 difficult.

Studies found gender differences, with female GPs reporting worse outcomes on all facets of psychological wellbeing.^30^^,^^36^^,^^38^^,^^39^^,^^41^^,^^47^^,^^48^ Similar findings have been reported in other physician groups in China,^60^ and greater job strain has been reported among female doctors in dual-doctor marriages during the pandemic.^61^ Experiences according to age varied across studies, with higher stress reported in older groups but more anxiety and depression in younger groups.

Studies included in this review highlight GPs’ plans to leave medicine,^30^^,^^31^^,^^44^ both to protect family members from risk of infection and because of the effects on their psychological wellbeing. Understanding the key sources of stress for GPs could enable an evidence-based approach to the development of future policy as the pandemic progresses, which may help to protect the future wellbeing of the workforce.

### Strengths and limitations

To the authors’ knowledge, this is the first systematic review exploring GPs’ psychological wellbeing during the COVID-19 pandemic. A rigorous methodology was used, and the combination of qualitative and quantitative literature generates an in-depth understanding of stressors and outcomes. Issues faced during the first year of the pandemic may be over-represented because of time-lags in publishing studies; further research may be needed to explore later experiences. There are limitations to these findings relating to their context, for example, non-English language studies were excluded.

Although the quality of the evidence was generally good, there were some limitations in consideration of confounders and in reporting across studies, with results pertaining to GPs often not disentangled from other healthcare workers, limiting the pool of research. Furthermore, most studies used cross-sectional survey designs so there may be selection bias in the types of GPs responding. The lack of longitudinal cohort designs limits the ability to assess the impact of the pandemic, and one study^55^ relied on participants’ retrospective judgement, which may be flawed because of potential recall bias. There is a need to standardise tools across studies, particularly around workplace stress and burnout.

### Comparison with existing literature

Although GP mental health and wellbeing has been the focus of a growing international debate, this current review is, to the authors’ knowledge, the first evidence synthesis on this topic.

Policymakers may wish to consider the strength of evidence from their particular settings, with potential need for further research reflecting variations in government and population responses to the pandemic, infection rates, and healthcare systems. For example, further research is needed from the US, India, and Brazil, which have had the highest absolute numbers of confirmed COVID-19 deaths as of March 2022.^62^ To the authors’ knowledge, just two studies exist from the US,^34^^,^^57^ both focused only on the uptake of telemedicine during the pandemic. Although three studies included GPs in the UK, these were limited to one geographical area,^55^ focused on GPs’ experiences of long COVID,^53^ or formed part of international evidence from different settings.^56^ Further UK evidence is needed.

### Implications for research and practice

COVID-19 has presented many challenges and created additional pressures for the GP workforce. The present study provides an international overview of the sources of stress and psychological outcomes, and highlights the need for policy and practice to support GPs.

Gender and age differences are noteworthy and may warrant further exploration. Although women may be more open in discussing difficulties and seeking support because of socialised gender norms,^63^ women may also have experienced greater pressures during the pandemic because of wider caring responsibilities.^61^ Increasing stress with age may result from seniority and additional roles including practice management. Policymakers and researchers may wish to consider these gender and age differences to design tailored interventions. Despite the increased risk of COVID-19 among some ethnic minority groups in the UK,^64^ there was a lack of evidence exploring the impact of ethnicity on measures of psychological wellbeing.

This review of international evidence demonstrates that the COVID-19 pandemic has adversely affected GPs’ wellbeing around the world. Policy and infrastructure are needed to support GPs during this challenging time.

## Data

Materials and data used for the conduct of this research are available from the study authors on request.
